# Supplementary material for: The past, present and future impact of HIV prevention and control on HPV and cervical disease in Tanzania: A modelling study
Source: PLoS One. 2020 May 6;15(5):e0231388. doi: 10.1371/journal.pone.0231388 (PMC7202618; doi:10.1371/journal.pone.0231388)
Supplement: S1 File — (PDF) [file pone.0231388.s001.pdf]

Supplementary appendix to the article:

The past, present and future impact of HIV prevention and control on HPV and cervical disease in Tanzania: a modelling study

Michaela T. Hall[1,2*], Megan A. Smith[2,3], Kate T. Simms[2,3], Ruanne V. Barnabas[4], Karen Canfell[2,3] and John M. Murray[1]

1. School of Mathematics and Statistics, UNSW Sydney, Australia
2. Cancer Research Division, Cancer Council NSW, Australia
3. School of Public Health, University of Sydney, Australia
4. University of Washington, Seattle, WA, USA

* Corresponding author: michaela.hall@nswcc.org.au

Additional equations for calculating the force of infection

Following on from the calculation of the force of infection for HIV and HPV 16/18 contained in the main manuscript, the force of infection of HPV H5 and HPV OHR, for high activity males aged a at time t are calculated using the below equations s1 to s4. Recall that HPV H5 refers to HPV types 31, 33, 45, 52 and 58, whereas HPV OHR refers to all oncogenic HPV types excluding HPV 16/18 and HPV H5.

$\Lambda_M^{HPV_{H5}}(a,r,t) =$

$$c_M(a,r,t)\big(1-\kappa_{HPV}(t)\big)\big(1-\upsilon_{HPV}(t)\big)T_{FM}^{HPV_{H5}}\left(\rho_M(a)\cdot \mathrm{I}_{\mathrm{F}}^{\mathrm{HPV}_{H5}}(t)\right)+\lambda_M^{HPV_{H5}}(a,r,t) \qquad (s1)$$

$\lambda_M^{HPV_{H5}}(a,r,t) =$

$$\varsigma_M(a,r,t)\big(1-\kappa_{HPV}(t)\big)\big(1-\upsilon_{HPV}(t)\big)T_{FM}^{HPV_{H5}}\left(\rho_M(a)\cdot \mathrm{I}_{\mathrm{CSW}}^{\mathrm{HPV_{H5}}}(t)\right) \qquad (s2)$$

$\Lambda_M^{HPV_{OHR}}(a,r,t) =$

$$c_M(a,r,t)\big(1-\kappa_{HPV}(t)\big)\big(1-\upsilon_{HPV}(t)\big)T_{FM}^{HPV_{OHR}}\left(\rho_M(a)\cdot \mathrm{I}_{\mathrm{F}}^{\mathrm{HPV}_{OHR}}(t)\right)+\lambda_M^{HPV_{OHR}}(a,r,t) \qquad (s3)$$

$\lambda_M^{HPV_{OHR}}(a,r,t) =$

$$\varsigma_M(a,r,t)\big(1-\kappa_{HPV}(t)\big)\big(1-\upsilon_{HPV}(t)\big)T_{FM}^{HPV_{OHR}}\left(\rho_M(a)\cdot \mathrm{I}_{\mathrm{CSW}}^{\mathrm{HPV_{OHR}}}(t)\right) \qquad (s4)$$

Here, $T_{FM}^{HPV_{H5}}$ and $T_{FM}^{HPV_{OHR}}$ are per sex-act female-to-male HPV H5 and HPV OHR transmission probabilities, and $I_F^{HPV_{H5}}(t)$ and $I_F^{HPV_{OHR}}(t)$ are vectors specifying the age-specific probability of a female being HPV H5 and HPV OHR positive, respectively.

We can define the force of infection for females in a similar way as for males; except that we have no need to account for the protective effects of VMMC, and we consider HIV acquisition among commercial sex workers from their male clients. Therefore, we define the force of HIV, HPV16/18 and HPV OHR infection in females as follows:

$\Lambda_F^{\mathrm{HIV}}(a,r,t) =$

$$c_F(a,r,t)\big(1-\kappa_{HIV}(t)\big)\sum_i\left(\sum_{Tx}\left(\rho_F(a)\cdot T_{MF}^{HIV(Tx)}(i)\right)I_M^{HIV(Tx)}(i,t)\right)+\lambda_F^{\mathrm{HIV}}(a,r,t) \qquad (s5)$$

$\lambda_F^{\mathrm{HIV}}(a,r,t) =$

$$\varsigma_F(a,r,t)\big(1-\kappa_{HIV}(t)\big)\sum_i\left(\sum_{Tx}\left(\rho_F(a)\cdot T_{MF}^{HIV(Tx)}(i)\right)I_{Client}^{HIV(Tx)}(i,t)\right) \qquad (s6)$$

$\Lambda_F^{HPV_{1618}}(a,r,t) =$

$$c_F(a,r,t)\big(1-\kappa_{HPV}(t)\big)T_{MF}^{HPV_{1618}}\left(\rho_F(a)\cdot \mathrm{I}_M^{\mathrm{HPV}_{1618}}(t)\right)+\lambda_F^{HPV_{1618}}(a,r,t) \qquad (s7)$$

$\lambda_F^{HPV_{1618}}(a,r,t) =$

$$\varsigma_F(a,r,t)\big(1-\kappa_{HPV}(t)\big)T_{MF}^{HPV_{1618}}\left(\rho_F(a)\cdot \mathrm{I}_{Client}^{\mathrm{HPV}_{1618}}(t)\right) \qquad (s8)$$

$\Lambda_F^{HPV_{H5}}(a,r,t) =$

$$c_F(a,r,t)\big(1-\kappa_{HPV}(t)\big)T_{MF}^{HPV_{H5}}\left(\rho_F(a)\cdot \mathrm{I}_M^{\mathrm{HPV}_{H5}}(t)\right)+\lambda_F^{HPV_{H5}}(a,r,t) \qquad (s9)$$

$\lambda_F^{HPV_{H5}}(a,r,t) =$

$$\varsigma_F(a,r,t)\big(1-\kappa_{HPV}(t)\big)T_{MF}^{HPV_{H5}}\left(\rho_F(a)\cdot \mathrm{I}_{Client}^{\mathrm{HPV}_{\mathrm{H5}}}(t)\right) \qquad (s10)$$

$\Lambda_F^{HPV_{OHR}}(a,r,t) =$

$$c_F(a,r,t)\big(1-\kappa_{HPV}(t)\big)T_{MF}^{HPV_{OHR}}\left(\rho_F(a)\cdot \mathrm{I}_M^{\mathrm{HPV}_{OHR}}(t)\right)+\lambda_F^{HPV_{OHR}}(a,r,t) \qquad (s11)$$

$\lambda_F^{HPV_{OHR}}(a,r,t) =$

$$\varsigma_F(a,r,t)\big(1-\kappa_{HPV}(t)\big)T_{MF}^{HPV_{OHR}}\left(\rho_F(a)\cdot \mathrm{I}_{Client}^{\mathrm{HPV}_{\mathrm{OHR}}}(t)\right) \qquad (s12)$$

The parameter definitions for the female force of infection equations (s5-s12) are translatable from their male equivalents, with the exception of $\varsigma_F(a,r,t)$, which denotes the per-timestep volume of clients for commercial sex workers (this is assumed to be zero for women who are not commercial-sex-workers).

Detailed parameter inputs relating to sexual behaviour and force of infection

Model input parameter assumptions specific to sexual behaviour and force of infection are summarised in Table S1. We define the following equations which are referred to in Table S1:

$$y = \lambda e^{-\left(\frac{x-\mu}{\sigma}\right)^2} \qquad (s13)$$

$$y = \frac{a_1 x - a_2}{b_1 x^2 - b_2 x + b_3} \qquad (s14)$$

Table S1 Model parameters relating to sexual behaviour in Tanzania.

**Parameter Description**	**Parameter Value**	**Data Source and/or Rationale**
Commercial sex worker population size and distribution.	1% of females aged 15-54 are sex workers. These 1% are broken down into the following age-groups: 36% of CSW are aged 15-24 years, 40% are 25-34 years, 22% are 35-49 years and 2% are 50-54 years.	The total CSW population size is based on UNAIDS 2018 update (UNAIDS estimate of 155,450 CSWs in 2014)[1]. The age-distribution of CSW's was based on the NACP report on Female Sex Workers in Dar es Salaam (2010)[2].
Age-specific per timestep CSW client volume. Used to calculate $\varsigma_F(a,r,t)$.	Normally distributed around a mean age of 39 for female sex workers aged 15-49 years. Defined by equation s$s13$ where x is equal to the youngest age in each specified age group. Here, $\lambda_{CSW} = 20$, $\mu_{CSW} = 39$ and $\sigma_{CSW} = 42$.	This is based on the age-distributions provided in the NACP report on FSW in Dar es Salaam, in addition to qualitative data which indicates that on average, Tanzanian FSWs have 26 clients per month, as reported in qualitative data [2, 3].
Age-specific per-timestep probability of a male paying for commercial sex. Used to calculate $\varsigma_M(a,r,t)$.	Specified by equation s14 for males aged 15 years and over where x is equal to the youngest age in the specified age group, and $a_1 = 0.5294$, $a_2 = 7.608$, $b_1 = 1$, $b_2 = 23.34$ and $b_3 = 153.6$.	This was based on fitting the age-specific probability of a male having paid for sex in the last 12 months, as published in the 2007-08 HIV/AIDS and Malaria indicator survey [4].
Probability of condom usage for commercial sex interactions. This is used to calculate $\kappa_{HIV}(t)$ and $\kappa_{HPV}(t)$.	Specified by equation s14 where x is equal to the calendar year (for 1993 onwards), and $a_1 = 1.01$, $a_2 = 2016$, $b_1 = 0$, $b_2 = 1$ and $b_3 = 1987$.	Parameter equation specified to fit survey data on condom use among commercial sex interactions[4-6].

Parameter Description	Parameter Value	Data Source and/or Rationale
Age-specific probability of males being in the high-activity sexual activity group.	The age-specific probability of a male being in the high-activity groups are as follows:<br>15-19 years: 0.05<br>20-24 years: 0.13<br>25-44 years: 0.12<br>45+ years: 0.03	Specified for consistency with the observed data (Munguti et al)[7]. The 'high-activity' group is equivalent to having 5+ sexual partners in a 12-month period as published in Munguti et al.
Age-specific probability of females being in the high-activity sexual activity group (excludes commercial sex workers).	The age-specific probability of a female being in the high-activity groups are as follows:<br>15-24 years: 0.03<br>25+ years: 0.01	Specified for consistency with the observed data (Munguti et al)[7]. Note that the probability of being in the high-risk sexual activity group for females aged 25 years and over was modified from zero to one percent. The 'high-activity' group is equivalent to having 5+ sexual partners in a 12-month period as published in Munguti et al.
Volume of sexual interactions possibly resulting in HIV transmission per timestep for high-activity males and females $c_M(a,1,t)$ and $c_F(a,1,t)$. Note that $r=1$ for high-activity individuals.	Normally distributed around a mean age of 30 for males and 17 for females. Defined by equation $s1$ where x is equal to the youngest age in each specified age group.<br>For males: $\lambda_{HAM}=162$, $\mu_{HAM}=30$ and $\sigma_{HAM}=19$. And for females: $\lambda_{HAF}=294$, $\mu_{HAF}=17$ and $\sigma_{HAF}=6$.<br>Note that λ_{HAM} and λ_{HAF} are varied in sensitivity analysis.	Determined via calibration algorithm.
Volume of sexual interactions possibly resulting in HIV transmission per timestep for general-activity males and females $c_M(a,0,t)$ and $c_F(a,0,t)$. Note that $r=1$ for general-activity individuals.	Normally distributed with mean age of 30 for males and 28 for females. Defined by equation $s1$ where x is equal to the youngest age in each specified age group.<br>For males: $\lambda_{GAM}=9.45$, $\mu_{GAM}=30$ and $\sigma_{GAM}=12$. And for females: $\lambda_{GAF}=9.50$, $\mu_{GAF}=28$ and $\sigma_{GAF}=6.8$.<br>Note that λ_{GAM} and λ_{GAF} are varied in sensitivity analysis.	Determined via calibration algorithm.

Parameter Description	Parameter Value	Data Source and/or Rationale
Probability of condom use for non-commercial sex interactions and condom efficacy. This is used to calculate $\kappa_{HIV}(t)$ and $\kappa_{HPV}(t)$.	Specified by a smoothed piece-wise linear interpolant from 0 in 1993, to 0.55 in 2011, to 0.37 in 2016 and constant thereafter. Therefore $\kappa_{HIV}\ (t) = p_{CUse}$ and $\kappa_{HPV}(t) = p_{CUse} \times 0.46$ for males and $\kappa_{HPV}(t) = p_{CUse} \times 0.3$ for females.	Specified to fit survey data on condom use among non-commercial sex interactions [4-6, 8-10].
Year-specific reduction in the number of high-risk sexual interactions among both sexes and all activity groups. Parameter reflects nationwide behavioural change in response to the HIV epidemic.	Specified by equation s13 where x is equal to the calendar year between 1995 and 2010, and $\lambda_{SB} = 0.5$, $\mu_{SB} = 2002$, $\sigma_{SB} = 8$. From 2010 onwards, this function is set to a constant value of 0.05.	Determined via calibration algorithm. This parameter reflects the behavioural change in Tanzania in response to public fear of HIV infection at the peak of the AIDS epidemic.

For each female age, the age-distribution of male partners is determined by the probability density function of the Poisson distribution over a maximum of five age groups (her current age group and the four above her if possible), with a Poisson parameter decreasing linearly with age from some maximum λ_{max} to 0 (which refers to the next five-year age group). The baseline value of this maximum is 1.5 (i.e. this is an average of 7.5 years older than the female age). This was based on observed data indicating that males tend to have sex with females younger than themselves[7]. This matrix is utilised to determine $\rho_M(a)$ and $\rho_F(a)$ for use in the force of infection calculation.

Model input parameters for the per-sex-act transmission probability is given in Table S2. These parameters reflect the variability in the probability of transmission among HIV sero-discordant couples, depending on the stage of disease of the HIV positive partner. The stage-specific per-contact transmission probabilities were sourced from Quinn et al[11].

Table S2 Stage-specific per-contact probability of HIV transmission.

	**Acute infection**	**WHO clinical stage 1**	**WHO clinical stage 2**	**WHO clinical stage 3**	**WHO clinical stage 4 (AIDS)**
**Transmission multipliers against base HIV transmission probability (0.0006)⁺**	$T_{FM}^{HIV(0)}(1) = 0.0006 \times 7$	$T_{FM}^{HIV(0)}(2) = 0.0006$	$T_{FM}^{HIV(0)}(3) = 0.0006 \times 5.8$	$T_{FM}^{HIV(0)}(4) = 0.0006 \times 6.8$	$T_{FM}^{HIV(0)}(5) = 0.0006 \times 11.8$

⁺Note that these probabilities are for female to male transmission; for male to female transmission we note that $T_{MF}^{HIV(Tx)} = 2.5 \times T_{FM}^{HIV(Tx)}$; the probabilities assume no ART treatment $Tx = 0$, during contacts with treated individuals $Tx = 1$ we note that the transmission probability is always reduced by 0.96.

Type-specific per high-risk-sex-act transmission probabilities are given in Table S3. These values were found through calibration. Burchell et al 2011 indicate that the probability of HPV transmission among sero-discordant general partnerships is 20% over a six month period[12].

Table S3 Transmission probabilities per high-risk sexual interaction for HPV 16/18, HPV 31/33/45/52/58 and other oncogenic HPV

**Parameter***	**Value**	**Probability of transmission over a six-month period⁺**
HPV 16/18 transmission probability per high-risk-sex-act $T_{FM}^{HPV_{1618}}$	0.0560	46% - 76%
HPV 31/33/45/52/58 transmission probability per high-risk-sex-act $T_{FM}^{HPV_{H5}}$	0.0123	14% - 47%
Other oncogenic high-risk HPV $T_{FM}^{HPV_{OHR}}$	0.0179	19% - 57%

** Note that $T_{FM}^{HPV} = T_{MF}^{HPV}$ for all HPV types. ⁺Calculated for males and females in the general activity group assuming condom use and efficacy (46% for males and 30% for females) as observed in 2011; ranges provided reflect variation in sexual frequency due to age; number included for comparison with Burchell et al 2011 [12].*

Table S4 HPV-type specific multipliers for acquisition, progression and regression of HPV associated disease for HIV positive individuals(11).*

**Parameter description**	**Parameter value⁺**
Multiplier increasing HPV acquisition for HIV positive individuals	2.75 (1.17-2.75)
Clearance of an HPV infection (no CIN)	0.6
Progression from HPV infection to CIN1	3.73 (2.62-3.73)
Progression from HPV infection to CIN2	1.3 (1.1-1.33)

Regression from CIN1 to clearance or HPV infected	0.7 (0.56-0.7) for HIV 16/18<br>0.67 (0.56-0.67) for all other HPV types.
Regression from CIN2/3 to clearance, HPV infection or CIN1	0.57 (0.26-0.57)
Progression from CIN3 to invasive cervical cancer	2.5 (2.3-2.5)

* *Please note that the ranges specified are those considered in sensitivity analysis, for information please refer to the following section titled 'Sensitivity analysis'.*

+*Note that these parameters are the same for all HPV types except where otherwise stated.*

Additional tables

The natural history transition probabilities for HPV infection are adapted from previously published models (see Tan et al 2018 supplementary appendix Table S25 a)[13].

Table S5 Per-timestep disease transition probabilities for HPV 16/18 infections and associated disease, by age, in HIV negative women.

**Age**	**From HPV infected to:**					**From CIN 1 to:**					**From CIN 2 infected to:**					**From CIN 3 to:**					
	**Well+**	**HPV**	**CIN 1**	**CIN 2**	**CIN 3**	**Well+**	**HPV**	**CIN 1**	**CIN 2**	**CIN 3**	**Well+**	**HPV**	**CIN 1**	**CIN 2**	**CIN 3**	**Well+**	**HPV**	**CIN 1**	**CIN 2**	**CIN 3**	**Undet ICC**
**10-14**	0.2013	0.035	0.0569	0.0171	0	0.0684	0.0056	0.2844	0.0088	0.0088	0.0679	0.0066	0.0238	0.1613	0.0369	0	0	0.0134	0.0097	0.5	0
**15-19**	0.2013	0.035	0.0569	0.0171	0	0.0684	0.0056	0.2844	0.0088	0.0088	0.0679	0.0066	0.0238	0.1613	0.0369	0	0	0.0134	0.0097	0.5	0
**20-24**	0.2013	0.0349	0.0569	0.0171	0	0.0684	0.0056	0.2843	0.0088	0.0088	0.0679	0.0066	0.0238	0.1613	0.0369	0	0	0.0134	0.0097	0.5	0.001
**25-29**	0.1381	0.1222	0.0312	0.0068	0	0.0684	0.0056	0.2767	0.0102	0.0102	0.0679	0.0066	0.0238	0.1352	0.0532	0	0	0.0134	0.0097	0.5	0.0014
**30-34**	0.1381	0.1214	0.0312	0.0068	0	0.0684	0.0056	0.2751	0.0102	0.0102	0.0679	0.0066	0.0238	0.1343	0.0532	0	0	0.0134	0.0097	0.5	0.0033
**35-39**	0.1381	0.1246	0.0312	0.0034	0	0.0684	0.0056	0.2719	0.0102	0.0102	0.0679	0.0066	0.0238	0.1183	0.0646	0	0	0.0056	0.0097	0.5	0.0033
**40-44**	0.121	0.1403	0.0312	0.0034	0	0.0684	0.0056	0.2663	0.0102	0.0113	0.0679	0.0066	0.0238	0.1167	0.0646	0	0	0.0056	0.0038	0.5	0.0063
**45-49**	0.1052	0.1689	0.0241	0.0017	0	0.0684	0.0056	0.2527	0.0136	0.0113	0.0679	0.0066	0.0238	0.105	0.0723	0	0	0.0056	0.0038	0.5	0.0063
**50-54**	0.0906	0.1897	0.0362	0.0025	0	0.0684	0.0056	0.2544	0.0204	0.0169	0.0679	0.0066	0.0238	0.106	0.1085	0	0	0.0056	0.0028	0.5	0.0095
**55-59**	0.077	0.2114	0.041	0.0029	0	0.0684	0.0056	0.2554	0.0232	0.0191	0.0679	0.0066	0.0238	0.098	0.1363	0	0	0.0056	0.0028	0.5	0.015
**60-64**	0.0642	0.2344	0.0458	0.0032	0	0.0684	0.0056	0.2561	0.0259	0.0214	0.0679	0.0066	0.0238	0.0983	0.1523	0	0	0.0019	0.0019	0.5	0.0168
**65-69**	0.0642	0.2358	0.0506	0.0035	0	0.0684	0.0056	0.2576	0.0286	0.0236	0.0679	0.0066	0.0238	0.0909	0.1852	0	0	0.0019	0.0019	0.5	0.0186
**70-74**	0.0642	0.2341	0.0554	0.0039	0	0.0684	0.0056	0.2557	0.0314	0.0259	0.0679	0.0066	0.0238	0.0899	0.2028	0	0	0.0019	0.0019	0.5	0.0203
**75-79**	0.0642	0.2279	0.0603	0.0042	0	0.0684	0.0056	0.2699	0.0341	0.0281	0.0679	0.0066	0.0238	0.0783	0.2408	0	0	0.0019	0.0009	0.5	0.0253
**80-84**	0.0642	0.2153	0.0241	0.0017	0	0.0684	0.0056	0.2352	0.0136	0.0113	0.0679	0.0066	0.0238	0.071	0.0963	0	0	0.0019	0.0009	0.5	0.0101

+ *Well refers to per-timestep probability of clearance of HPV infection or CIN.*

Table S6 Per-timestep disease transition probabilities for high-risk HPV not 16/18 infections and associated disease, by age, in HIV negative women.

**Age**	**From HPV infected to:**					**From CIN 1 to:**					**From CIN 2 infected to:**					**From CIN 3 to:**					
	**Well^{+}**	**HPV**	**CIN 1**	**CIN 2**	**CIN 3**	**Well^{+}**	**HPV**	**CIN 1**	**CIN 2**	**CIN 3**	**Well^{+}**	**HPV**	**CIN 1**	**CIN 2**	**CIN 3**	**Well^{+}**	**HPV**	**CIN 1**	**CIN 2**	**CIN 3**	**Undet ICC**
**10-14**	0.3298	0.012	0.0334	0.01	0	0.0804	0.0097	0.2268	0.0051	0.0051	0.1143	0.0114	0.0407	0.0984	0.0203	0	0	0.023	0.0163	0.4044	0
**15-19**	0.3298	0.012	0.0334	0.01	0	0.0804	0.0097	0.2268	0.0051	0.0051	0.1143	0.0114	0.0407	0.0983	0.0203	0	0	0.023	0.0163	0.4042	0
**20-24**	0.3298	0.0119	0.0334	0.01	0	0.0804	0.0097	0.2267	0.0051	0.0051	0.1143	0.0114	0.0407	0.0983	0.0203	0	0	0.023	0.0163	0.4002	0.0006
**25-29**	0.2273	0.0764	0.0182	0.0039	0	0.0804	0.0097	0.2212	0.0059	0.0059	0.1143	0.0114	0.0407	0.087	0.0312	0	0	0.023	0.0163	0.3989	0.0008
**30-34**	0.2273	0.0757	0.0182	0.0039	0	0.0804	0.0097	0.22	0.0059	0.0059	0.1143	0.0114	0.0407	0.0841	0.0312	0	0	0.023	0.0163	0.3887	0.0019
**35-39**	0.2273	0.0767	0.0182	0.002	0	0.0804	0.0097	0.2175	0.0059	0.0059	0.1143	0.0114	0.0407	0.0769	0.038	0	0	0.0097	0.0163	0.5	0.0019
**40-44**	0.2	0.0923	0.0182	0.002	0	0.0804	0.0097	0.214	0.0059	0.0065	0.1143	0.0114	0.0407	0.0735	0.038	0	0	0.0097	0.0065	0.5	0.0036
**45-49**	0.1769	0.115	0.0141	0.001	0	0.0804	0.0097	0.2059	0.0079	0.0065	0.1143	0.0114	0.0407	0.066	0.0426	0	0	0.0097	0.0065	0.5	0.0036
**50-54**	0.1512	0.1366	0.0211	0.0015	0	0.0804	0.0097	0.2072	0.0119	0.0098	0.1143	0.0114	0.0407	0.0668	0.0639	0	0	0.0097	0.0069	0.5	0.0053
**55-59**	0.1291	0.1601	0.0239	0.0017	0	0.0804	0.0097	0.2081	0.0135	0.0111	0.1143	0.0114	0.0407	0.0623	0.1161	0	0	0.0097	0.0069	0.5	0.0085
**60-64**	0.1081	0.1862	0.0267	0.0019	0	0.0804	0.0097	0.2086	0.0151	0.0124	0.1143	0.0114	0.0407	0.0626	0.1298	0	0	0.0032	0.0032	0.5	0.0095
**65-69**	0.1081	0.1873	0.0295	0.0021	0	0.0804	0.0097	0.2099	0.0167	0.0137	0.1143	0.0114	0.0407	0.0584	0.1094	0	0	0.0032	0.0032	0.5	0.0105
**70-74**	0.1081	0.1859	0.0323	0.0022	0	0.0804	0.0097	0.2083	0.0182	0.015	0.1143	0.0114	0.0407	0.0577	0.1199	0	0	0.0032	0.0032	0.5	0.0115
**75-79**	0.1081	0.1808	0.0351	0.0024	0	0.0804	0.0097	0.2027	0.0198	0.0164	0.1143	0.0114	0.0407	0.0493	0.1426	0	0	0.0032	0.0016	0.5	0.0143
**80-84**	0.1081	0.1704	0.0141	0.001	0	0.0804	0.0097	0.1914	0.0079	0.0065	0.1143	0.0114	0.0407	0.0427	0.057	0	0	0.0032	0.0016	0.5	0.0057

$^{+}$ *Well refers to per-timestep probability of clearance of HPV infection or CIN.*

References

1. UNAIDS. UNITED REPUBLIC OF TANZANIA 2018 [2018-09-10]. Available from: http://aidsinfo.unaids.org/.
2. United Republic of Tanzania Ministry of Health and Social Welfare National AIDS Control Programme (NACP). HIV Behavioral and Biological Surveillance Survey Among Female Sex Workers in Dar es Salaam, 2010. Dar es Salaam: 2012.
3. Research International (on behalf of T-MARC). Women engaged in sex work and transactional sex in Tanzania. 2009.
4. Tanzania Commission for AIDS (TACAIDS), Zanzibar AIDS Commission (ZAC), National Bureau of Statistics (NBS), Office of Chief Government Statistician (OCGS), Macro International Inc. Tanzania HIV/AIDS and Malaria Indicator Survey 2007-2008. Dar es Salaam, Tanzania: 2008.
5. Tanzania Commission for AIDS (TACAIDS), Zanzibar AIDS Commission (ZAC), National Bureau of Statistics (NBS), Office of Chief Government Statistician (OCGS), ICF International. Tanzania HIV/AIDS and Malaria Indicator Survey 2011-2012. Dar es Salaam, Tanzania: 2013.
6. Ministry of Health CD, Gender, Elderly and Children (MoHCDGEC), , Ministry of Health (MoH), National Bureau of Statistics (NBS), Office of the Chief Government Statistician (OCGS), ICF. Tanzania Demographic and Health Survey and Malaria Indicator Survey 2015-16. 2016.
7. Munguti K, Grosskurth H, Newell J, Senkoro K, Mosha F, Todd J, et al. Patterns of sexual behaviour in a rural population in north-western Tanzania. Soc Sci Med. 1997;44(10):1553-61. Epub 1997/05/01. PubMed PMID: 9160444.
8. Kapiga SH, Lugalla JL. Male condom use in Tanzania: results from a national survey. East Afr Med J. 2003;80(4):181-90. Epub 2003/08/16. PubMed PMID: 12918800.
9. Adair T. Men's condom use in higher-risk sex: Trends in five sub-Saharan African countries. Journal of Population Research. 2008;25(1):51-62. doi: 10.1007/bf03031940.
10. Reynolds HW, Luseno W, Speizer IS. The Measurement of Condom Use in Four Countries in East and Southern Africa. AIDS and behavior. 2012;16(4):1044-53. doi: 10.1007/s10461-012-0146-9. PubMed PMID: PMC3743219.
11. Quinn TC, Wawer MJ, Sewankambo N, Serwadda D, Li C, Wabwire-Mangen F, et al. Viral load and heterosexual transmission of human immunodeficiency virus type 1. Rakai Project Study Group. The New England journal of medicine. 2000;342(13):921-9. Epub 2000/03/30. doi: 10.1056/nejm200003303421303. PubMed PMID: 10738050.
12. Burchell AN, Coutlée F, Tellier P-P, Hanley J, Franco EL. Genital transmission of human papillomavirus in recently formed heterosexual couples. J Infect Dis. 2011;204(11):1723-9. Epub 2011/10/07. doi: 10.1093/infdis/jir644. PubMed PMID: 21984739.
13. Tan N, Sharma M, Winer R, Galloway D, Rees H, Barnabas RV. Model-estimated effectiveness of single dose 9-valent HPV vaccination for HIV-positive and HIV-negative females in South Africa. Vaccine. 2018;36(32 Pt A):4830-6. Epub 2018/06/13. doi: 10.1016/j.vaccine.2018.02.023. PubMed PMID: 29891348; PubMed Central PMCID: PMCPMC6508597.
